# “Just do it tomorrow”: Procrastination in Tunisian university students

**DOI:** 10.1192/j.eurpsy.2021.2014

**Published:** 2021-08-13

**Authors:** M. Ben Alaya, U. Ouali, E. Bouguira, Y. Zgueb, S. Ben Youssef, F. Nacef

**Affiliations:** 1 Department Psychiatry A, Razi Hospital, Manouba, Tunisia; 2 Nih, National Institute of Health, Tunis, Tunisia

**Keywords:** Impulsivity, Perfectionism, quality of life, Procrastination

## Abstract

**Introduction:**

Procrastination is a common phenomenon among students, defined by the tendency to delay tasks. Procrastination can have a negative impact not only on academic achievement but also on other areas of life.

**Objectives:**

To assess students’ level of procrastination and associated psychological factors

**Methods:**

We conducted a cross-sectional descriptive study in students from three different universities: a medical school, a law school and an engineering school. Socio-demographic, clinical and academic data were collected. Procrastination was assessed using the Pure Procrastination Scale. We further administered the Short Version of the impulsive behaviour scale, the Satisfaction with Life Scale, the Perfectionism scale, and the one item Self-esteem Scale.

**Results:**

Our sample consisted of 1019 students. The mean age was 22 ± 2.25 years, 62% were females and almost 70% were single. The mean level of procrastination was 35 ± 10.42. Procrastination was positively correlated with impulsivity (r= 0.37 p=0.00) and perfectionism (r= 1.32 p= 0.00) and negatively correlated with life satisfaction (r= -0.22 p = 0.00) and self-esteem (r= - 0.12 p= 0.00).

**Conclusions:**

The level of procrastination was relatively high in our study population. As described in the literature, impulsiveness and perfectionism were closely and positively related to procrastination, whereas higher procrastination scores were linked to lower quality of life and self-esteem. Our findings underline the need for counselling services with a focus on procrastination for university students.

**Disclosure:**

No significant relationships.

